# Impact of white matter hyperintensities on disease progression in Progressive Supranuclear Palsy‐Richardson syndrome

**DOI:** 10.1002/alz70856_107210

**Published:** 2026-01-08

**Authors:** Indira Ruth Garcia Cordero, Juan‐Camilo Vargas‐González, Blas Couto, Ece Bayram, Federico Rodriguez‐Porcel, Jay Iyer, Lawrence I Golbe, Christopher Stephen, Alex Pantelyat, Marian Dale, Nikolaus McFarland, Tao Xie, Matthew Swan, Kyurim Kang, Douglas Gunzler, Anne‐Marie Wills, Adam L. Boxer, Irene Litvan, Anthony E. Lang, Carmela Tartaglia

**Affiliations:** ^1^ Universidad de San Andres, Victoria, Buenos Aires, Argentina; ^2^ Tanz Centre for Research in Neurodegenerative Diseases, University of Toronto, Toronto, ON, Canada; ^3^ Memory Clinic, Toronto Western Hospital, University Health Network, Toronto, ON, Canada; ^4^ University Health Network, Toronto, ON, Canada; ^5^ Laboratory of Progressive Neuromotor and Neurobehavioral Disorders at Instituto de Neurociencia Cognitiva y Traslacional (INCYT), CABA, Buenos Aires, Argentina; ^6^ Instituto de Neurología Cognitiva (INECO), CABA, Buenos Aires, Argentina; ^7^ University of Colorado Denver, Aurora, CO, USA; ^8^ Medical University of South Carolina, Charleston, SC, USA; ^9^ Departments of Molecular and Cellular Biology and Statistics, Harvard University, MA, MD, USA; ^10^ Rutgers Robert Wood Johnson Medical School, New Brunswick, NJ, USA; ^11^ Department of Neurology, Massachusetts General Hospital and Harvard Medical School, Boston, MA, USA; ^12^ Johns Hopkins University School of Medicine, Baltimore, MD, USA; ^13^ Department of Neurology, Oregon Health & Science University, Portland, OR, USA; ^14^ Department of Neurology, Norman Fixel Institute for Neurological Diseases, College of Medicine, University of Florida, Gainesville, FL, USA; ^15^ Department of Neurology, University of Chicago Medicine, Chicago, IL, USA; ^16^ Department of Neurology, Mount Sinai Beth Israel, and Icahn School of Medicine, New York, NY, USA; ^17^ Music and Health Science Research Collaboratory (MaHRC), Faculty of Music, University of Toronto, Toronto, ON, Canada; ^18^ Population Health and Equity Research Institute, Center for Health Care Research and Policy, MetroHealth Medical Center, School of Medicine, Case Western Reserve University, Cleveland, Cleveland, OH, USA; ^19^ Massachusetts General Hospital, Boston, MA, USA; ^20^ Memory and Aging Center, Department of Neurology, Weill Institute for Neurosciences, University of California, San Francisco, San Francisco, CA, USA; ^21^ University of California, San Diego, La Jolla, CA, USA; ^22^ Rossy PSP Program, University Health Network and the University of Toronto, Toronto, ON, Canada; ^23^ Toronto Western Hospital, Toronto, ON, Canada; ^24^ Krembil Brain Institute, University Health Network (UHN), Toronto, ON, Canada

## Abstract

**Background:**

White matter hyperintensities (WMH) are recognized as neuroimaging biomarkers of cerebral small vessel disease; however, their clinical significance in Progressive Supranuclear Palsy‐Richardson syndrome (PSP‐RS) is poorly understood.

**Method:**

125 PSP‐RS patients from the Tilavonemab (ABBV‐8E12) clinical trial were assessed for disease severity using the PSP Rating Scale (PSPRS) at baseline and week 24. A PSPRS change index was calculated for week 24. WMH lesions were segmented on MRI FLAIR images using the Lesion Segmentation Tool and the total lesion volume (TLV) was calculated at baseline and week 24. The non‐laboratory Framingham Atherosclerotic Cardiovascular Disease Risk Score (FRS) was calculated in 99 patients. A linear mixed‐effect model for repeated measures was used to analyzed the TLV across the two time points. A multiple linear regression analyses was performed to analyze the association between baseline TLV, FRS, and their interaction in predicting the PSPRS change index. All analyses were adjusted for age, sex and disease duration.

**Result:**

Mean age of the 125 PSP‐RS patients: 68.7 (49‐86) years, 51 females (40.8%). There was an increase of the TLV (B=0.09, 95% Confidence Intervals (CI):0.04‐0.13, *p* <0.001; mean±SD: 8.46±10.20 vs 9.38±10.60 ml) across time. Mean age of the 99 PSP‐RS patients with FRS: 66.8 (49‐74) years, 43 females (43.4%). Mean FRS: 20.6 ± 8.3%. A significant relationship was found between the TLV and the PSPRS change index (B=0.08, CI:0.01‐0.15, *p* = 0.02) and between the FRS and the PSPRS change index (B=0.11, CI:0.01‐0.21, *p* = 0.03). TLV*FRS shows a significant negative association with the PSPRS change index (B=‐0.01, CI:‐0.01‐ 0.00, *p* = 0.02).

**Conclusion:**

TLV increased over time in PSP‐RS patients. For each 1 ml increase in TLV, the PSPRS change score increased by 0.11, and for each 1% increase in FRS, the PSPRS change score increased by 0.08. However, the increase must be adjusted for the combined effect, i.e., when both FRS and TLV are present, the increase is slightly smaller than the sum of their individual effects.

*Based on research using data from AbbVie that has been made available through Vivli, Inc. Vivli has not contributed to or approved, and is not in any way responsible for, the contents of this publication.

